# Much Improved and Extended

**Published:** 1850-04

**Authors:** 


Much improved and extended. *The Medical Formulary, being a collection of prescriptions derived from the writings and practice of many of the most eminent physicians in America and Europe; to which is added an Appendix, containing the usual dietetic preparations and antidotes for poisons: the whole accompanied with a few brief pharmaceutical and medical observations.* By BENJAMIN ELLIS, M. D., Late Professor of Materia Medica and Pharmacy in the Philadelphia College of Pharmacy. Morbos autem non eloquentiâ sed remediis curari.—Cels. de med. lib. i. Ninth edition, corrected and extended by SAMUEL GEORGE MORTON, M. D. 8vo. pp. 267. Philadelphia, 1849.

Were we to form a judgment of the relative extent of the two works before us by the length of the titles, we should be led into a grievous error; for there is no topic treated of in the latter, which is not contained in the former; whilst in the selection that has been made of the materials, more judgment has been displayed by Dr. Griffith; and there is a copiousness in his work, which we may look for in vain in his more showy companion and predecessor. In his motto "*selecta sunt quæ medicum nobilitant,*" from Linnæus, there is contained a truth too much overlooked, especially by those who are perpetually straining after originality, and are never satisfied unless novel assertions, and startling propositions, are brought forward. How much of our most instructive medical literature consists of the "*selecta*" judiciously made, and attractively presented! In a combination of art and science, which has existed from primeval periods; which is founded on an accumulation of observations and deductions—many carefully, others carelessly made by observers and reasoners in all ages—it is as arrogant as it would be impossible for any writer to attempt to form the science *de novo;* and to discard all that had been done by distinguished predecessors and contemporaries. To attempt, for example, to compose an original practice of medicine or an original treatise on the materia medica would be an absurdity. If it were *new,* it could not be a true exponent of the existing condition of a progressive science; whilst it would be a monument of the folly and presumption of its author. Hence it is, that the most valuable works of science, at the present day, are such as have brought together the "*selecta*" from the recorded observations and reflections of all periods. The mass requires to be sifted; the valuable ore to be separated, and the dross to be rejected; and no little skill and judgment is required on the part of him who undertakes the task.

The composer of a formulary belongs to the class of industrious laborers who are esteemed to be engaged in facilitating the onward progess of the *science* or rather of the *art* of medicine; and much of their success must depend upon the mode in which their "*selecta*" are made. We have had strong doubts, however, whether as much benefit has resulted from such works as their zealous authors may have imagined. The fact of one of those before us having passed through *eight editions* is sufficient evidence that they are popular—not on the principle, doubtless, embraced in the motto prefixed to it—"*Morbos autem non eloquentiâ sed remediis curari*"—for by most persons this would be considered a truism; but because they spare the prescriber the trouble of *thinking.* He sees a formula composed for a special malady, and employed by some eminent practitioner—if not eminent in reality, eminent by courtesy—adopts it unhesitatingly; learns to swear in *verba magistri,* and becomes gradually, if not at once, a mere routinist.

We apprehend, that this evil result from formularies is more common than it may seem to be at first sight. There is, in the young practitioner more especially, too great a degree of confidence in the power of medicine,—in the special adaptation of drugs to special morbid conditions: he fancies that he can cure diseases in many cases where his endeavors are impotent, and his interference meddlesome, and perhaps injurious; and he is apt to become the creature of combinations; and to be a drag on the onward course of his profession: for if there be any thing that more than another weighs on the progress of the science and art of medicine—is fatal indeed to its advancement—it is undue confidence in remedial agents; and defective acquaintance with therapeutical principles. We did not, indeed, require the pages of the works before us to demonstrate how few physicians there are who belong to the *viri prudentes* of Gaubius. "*Medicus vir prudens nihil præscribat nisi cujus sufficientem queat reddere rationem quum requiritur: hinc nunquam tumultuario, sed semper ex indicatione prius ritè deducta agat.*"

The "Formulary of Ellis" has been so long before the public, that we need not attract attention to it much farther than by stating, that the editor in preparing the present edition "has been at great pains to render the work in all respects deserving of continued professional confidence. The utmost care has been bestowed in correcting and extending its several parts, omitting some formulæ and inserting others, and bringing the whole up to the present state of pharmaceutical knowledge." Yet we think the pruning knife might have been employed more extensively, and without detriment to the work. It would be difficult, indeed, to comprehend the "principles" of combination which have prevailed in the throwing together—we know no better expression—of the heterogeneous assemblage of ingredients in some of the formulæ. As one of the unnecessary preparations we may cite the following:

*Enema with Tartrate of Antimony.*

℞. Antimonii et potassæ tartratis, ℈j to ℈ij,
Solutionis acaciæ tepidæ, Oj. Misce.

This preparation is stated to be "one admirably calculated to overcome constipation which resists the ordinary remedies." We have sundry objections, however, to it. In the first place, the title is defective. It should be "*Enema with tartrate of antimony and potassa*;" in the second, in a Latin formula ℈j. to ℈ij. ought to be ℈j. ad ℈ij.; and in the third, *Solutionis acaciæ tepidæ* conveys no definite idea to the young prescriber or to any one. We presume it means "tepid solution of acacia or gum arabic:" but, as to the strength, we are left wholly in the dark; admitting, however, the formula to be accurate in language, and intelligible in enunciation, it surely cannot be regarded as worthy of a distinct place in a formulary;—yet it is in that of Dr. Griffith also.

As an instance of heterogeneous combination—*farrago* would be too harsh a term for it—we may give the following 'expectorant' mixture :—

| | |
|---|---|
| " ℞. Tincturæ tolutani, | f.℥iss. |
| Acidi sulphurici aromatici, | f.ʒss. |
| Tincturæ digitalis, | f.ʒj. |
| Vini antimonii, | f.ʒij. |
| Mellis despumati, | f.℥iss. |
| Pulveris glycyrrhizæ, | ʒss. |
| Aquæ distillatæ, | f. ℥vj. |

Fiat mistura."

In this formula we have one *excitant expectorant*—tincture of tolu; two *demulcent expectorants*, honey and powdered liquorice;—[why *powdered* liquorice when we have the *extract?*]—one *pure sedative*—tincture of digitalis ; a grateful *acid*, but why introduced we know not; the aromatic sulphuric acid; and a *sedative* diaphoretic, the vinum antimonii ;—but the merest tyro in chemistry is aware, that sulphuric acid and tartrate of antimony and potassa are incompatible in the same prescription ; and that the author of the combination, who manifestly supposed he would have the effects of both, could not possibly have those of either; but of a compound of whose very existence he was in unquestioned ignorance. The formula is little or not at all more philosophical than the administration of sulphuric acid and acetate of lead at the same time, but in separate vehicles, in a case of hemorrhage—used, as we have known it, under the idea that each agent, singly, being astringent, when united they must necessarily be more efficacious! Not an idea was entertained by the blissful ignoramus, that an insoluble and inert sulphate of lead was formed, which could not possibly have any therapeutical agency.

But we have said as much as our space will permit of the 'Formulary' of Ellis ; and will only add, that the nomenclature has been improved ; and made to approximate more to that of the Pharmacopœia of the United States. There is still room, however, for farther improvement in this respect. For example : *Infusum* is now appropriately used for the product of *infusio* or the act of infusing; and hence we say *infusum sennæ*, not *infusio sennæ*, (p. 50;) as *præparatum* is employed for the thing prepared; *præparatio* for the act of preparing. We would suggest likewise, that if we apply the term at all to the pubes of dolichos (mucuna)

it must be *spiculum*, not *spicula* (p. 108.) We know of no such word as *spicula* for a small spike or dart :—that *vitellis* unius ovi (p. 144,) should be *vitellum ;* and in like manner *vitellum* ov. ij. should be *vitella.* The school master is now abroad in the profession, and these linguistic matters ought to be attended to, especially as books, like those before us, are more referred to by the younger than by the older practitioners.

The sphere embraced by the Formulary of Dr. Griffith—as the epithet 'universal' imports—is more extended. The design—as announced by the author—is "to present a compendious collection of formulæ and pharmaceutic processes, with such additional information as may render it useful to the physician and apothecary ; and the principal aim has been to select materials most generally applicable, and of practical utility. The sources from which they have been derived are very numerous, as will be seen by a reference to the various authorities cited." We wish that Dr. Griffith had given us the titles of the works from which his formulæ have been selected, rather than a mere allusion to their authors. The bibliography of formularies is rich and copious ; and our French and German brethren have been exceedingly prolific. Whether this be an evidence of their greater advancement in professional knowledge may admit of question. Our own mind has been long made up on this matter ; but others may not agree with us. We think, that where numerous formularies prevail, it is rather an evidence of a want of self-reliance, and *that*, perhaps, dependent upon imperfect knowledge, and an undue deference to the " words of the master."

A large amount of useful and selected matters, well arranged, with the original observations of the author interlarded, is made to precede and follow the Formulary proper: among these are the subject of weights and measures, domestic and foreign ; specific gravity ; temperatures ; vocabulary of words employed in prescriptions,—the less needed by the way, in the work before us, owing to Dr. Griffith's having adopted a course, which will be lauded by some and condemned by others, of giving all the ingredients of the formula in the vernacular ; observations on the management of the sick room ; doses of medicines ; rules for the administration of medicines ; management of convalescence and relapses; dietetic preparations; list of incompatibles; posological table; officinal preparations and directions; bloodletting; poisons; index of diseases and their remedies, &c. &c.

Dr. Griffith's formulary—unlike that of Dr. Ellis—is arranged alphabetically, "according to the pharmaceutic names adopted in the United States Pharmacopœia; but in each formula the English appellations for the articles composing it are used, and the quantities of these ingredients are expressed in words, and not in the usual pharmaceutic signs."

To a certain extent the objection already made to the formulary of Dr. Ellis, applies, however, to it. Numerous unnecessary and badly compounded formulæ are inserted; and a dangerous polypharmacy is encouraged, which the good sense of intelligent observers is now every where discarding. Imagine, for a moment, the rationale of such a preparation as the "*Compound Tincture of Opoponax*," (p. 304,) recommended by Brera, an Italian physician, as an application to foul venereal ulcers;—opoponax itself not being admitted into the Pharmacopœia of the United States; and not an allusion made to it in our dispensatories as an external remedy.

℞.* Round birthwort,
Long birthwort,
Orris root, each half an ounce,
Opoponax,
Sagapenum, each two drachms,
Guaiacum, four scruples,
Cloves, two drachms,
Camphor, three drachms,
Alcohol, ten ounces.

Macerate for twenty-four hours, and filter.

We plead guilty to consummate ignorance in regard to the principles of combination, if any such there be, that suggested this formula; and it is only one example of the endless permutations that might load the books, provided the crude notions of every prescriber were recorded. This, we doubt not, has received the name of 'Brera's Compound Tincture of Opoponax,' in the same way as the names of different respectable physicians have been

* If Dr. Griffith discards the technical Latin names in his formulæ, why does he retain the cloven foot of Jupiter, which now means *Recipe?*

attached to other formulæ for venal purposes. Aloes has been a god-send to the pill-inventors. A larger or a smaller proportion of the basis, with this or that adjuvant; and a variety of corrigents and excipients, has given occasion to the admission into the 'Formulary' under notice, of Anderson's, Barthez's, Becker's, Chapman's, Duchesne's, Frank's, Fuller's, Griffith's, Hooper's, James's, Lady Webster's, Mitchell's, Morrison's, Peter's, Pittschaft's, Speediman's, and Whytt's pills,—a motley assemblage of the names of eminent physicians, a titled lady, and numerous arrant empirics.

It is unfortunate that the names of respectable members of the profession should ever be permitted to be attached to such formulæ. How are the unprofessional to discriminate between the pills of Morrison the empiric, and of Hooper the respectable physician; and then the transition is so easy to the elixir of McMunn, the panacea of Swaim, and the drops of *Lignum*—for so an arch empiric of the name of *Wood* disguised his patronymic—that the landmarks between science and empiricism are destroyed, or overlooked; and a tendency is given to the latter even by those who would hesitate to prescribe a secret remedy, and be startled at the idea that they could be esteemed encouragers of quackery.

On the whole, it will be inferred, that we are not over-favorable to formularies; but if they are to exist—and we do not suppose that any prepossessions of ours will diminish their number—we think the one of Dr. Griffith is worthy of recommendation, not only on account of the care that has been bestowed upon it by its estimable author; but its general accuracy, and the richness of its details, which to him, however, who relies wholly upon it, can scarcely fail to prove *un embarras des richesses.*

